# Health Outcomes for Older Hispanics With HIV in New York City Using the Oaxaca Decomposition Approach

**DOI:** 10.5539/gjhs.v7n1p133

**Published:** 2014-08-22

**Authors:** Juan J. Dela Cruz, Stephen E. Karpiak, Mark Brennan-Ing

**Affiliations:** 1 CUNY–Lehman College and Graduate Center, 250 Bedford Park Blvd West, Bronx, NY, USA; 2 AIDS Community Research Initiative of America - Center on HIV and Aging, NYU-College of Nursing, 575 Eighth Avenue, Suite 502, New York, NY, USA

**Keywords:** HIV, older adults, Hispanics, depression and Oaxaca Decomposition

## Abstract

Although HIV and aging are two well-established medical and economic domains, their intersection represents an emerging area of study. Older adults with HIV, who sill comprise 50% of the US HIV-infected population by 2015, are disadvantaged as evidenced by disproportionately poorer health outcomes. The Oaxaca Decomposition Approach (ODA) was used to analyze data from the Research on Older Adults with HIV (ROAH) Study of 1,000 older adults with HIV in New York City (NYC). This paper establishes the sources of health disparities for Hispanics with HIV compared to a match group of Non-Hispanics with HIV. The ODA analyses shows that Hispanics on average have higher levels of declining health and increased depression attributable to the discrimination factor.

## 1. Introduction

Empirical evidence supports the views that sexual behaviors, race/ethnicity, psychosocial variables as well as epidemiological (risk associated with the acquisition and transmission of disease) and socioeconomic factors are related to HIV infection across individuals (DelaCruz, 2009). In the US, the HIV population is aging so that by 2015 half of the over 1.3 million people with HIV will be age 50 and older (Center for Disease Control and Prevention, CDC). Yet, the impact of HIV on aging populations and, in particular on aging minorities, is poorly understood. Hispanics/Latinos, namely people of Spanish culture regardless of race, are the fastest-growing racial/ethnic minority in the US and will represent 30% of the population by 2050. According to Avert (2013), Hispanics are disproportionately affected by HIV/AIDS. While Hispanics represent 17% of population, they account for 21% of new HIV infections, and 20% of those living with the virus. HIV infection in Hispanics is over three times that of Whites, and accounts for 20% of all new stage 3 AIDS diagnoses. Evidence shows significant health disparities exist for older Hispanics with HIV and their prevalence is five times greater for Hispanics 50 and older compared with non-Hispanic Whites (Linley, 2012). Unfortunately, policy makers and civil society underestimate the complexity and significance of adults aging with HIV (Emlet, 2004). It is likely that health disparities and poor health outcomes will be magnified as the HIV population ages, and will be the most acute among minority groups.

This paper examines HIV disparities among older Hispanics with HIV compared to Non-Hispanics with HIV using the Oaxaca Decomposition Approach. This topic is significant because the convergence of HIV and aging is an emergent area of study as the disease becomes more prevalent among people 50 years and older. HIV became a significant public health issue in the 1980’s, causing disability and death among individuals in their productive and reproductive years (DelaCruz, 2013). Hispanics are at increased risk of HIV infection due to pervasive stigma and discrimination. Those who survived during the first decade of the epidemic are now older adults who rely on panoply of health programs both private and publicly funded. Racial/ethnic minorities have disproportionately high infection rates and experience additional barriers accessing and receiving treatment, which can both cause and exacerbate other health disparities. HIV is now classified as a chronic and manageable disease. Sustained improvements in health status depend on rigorous antiretroviral therapy (ART) adherence and multimorbidity management. More economic analysis is needed to examine the determinants of HIV among older Hispanics and their health outcomes.

The Administration on Aging (2013) reported that demographic and epidemiological trends are rapidly transforming the population pyramid in the US so that nearly one-in-five adults will be age 65 or older by 2030. In parallel, the age distribution of the US HIV population is shifting rapidly. CDC surveillance data shows that people over the age of 50 with HIV (i.e., older adults) will account for 50% of the total HIV infected population by 2015, and will predictably rise to more than 70% by 2020 (CDC Surveillance Data, 2013). This aging of the HIV epidemic can be traced to one primary cause–the efficacy of ART introduced in the mid-1990s that produced a sharp decline in HIV morbidity and mortality. Before 1995 a person with a diagnosis of HIV/AIDS faced the likelihood of death within a few years if not months. Today a person diagnosed with HIV who adheres to ART can expect to live a near normal life span. A smaller contributor to the aging process of the HIV population, are those older adults who become infected with HIV after age 50.

Aging with HIV is a complex public health issue and therefore an economic issue (High, 2012). Older adults with HIV are being diagnosed with illnesses associated with advanced age decades earlier than their non-infected peers. Some speculate that HIV may contribute to an acceleration or accentuation of aging processes, as evidenced by high rates of cardiovascular disease, cancers, kidney disease, diabetes, osteoporosis, frailty and neurocognitive deficits among those aging with HIV, whose median age was 58 in 2010 (CDC Surveillance Data). The comorbidities experienced by this population are highly associated with elevated levels of depression (Havlik, 2011). Depression among older adults with HIV has been reported at five times the level in the general population and is fueled by stigma and resultant loneliness due to rejection and withdrawal (Emlet, 2006; Grov, 2010). The single most valid predictor of non-adherence to ART is depression (Psaros, 2009; Campos, 2010). Adherence to ART medications is unique. A lifetime rate of 90% adherence is required to sustain suppression of HIV replication thereby preventing the collapse of the immune system (Gonzalez, Psaros, Batchelder, Applebaum, Newville, & Safren, 2011). A 2011 meta-analysis of 95 studies representing over 36,000 patients with HIV shows a consistent relationship between depression and poor ART adherence (Gonzalez, Batchelder, Psaros, & Safren, 2011).

The aging population with HIV is a success complicated by unexpected health care challenges with significant socioeconomic consequences. The occurrence of multiple incurable chronic disorders, termed multi-morbidity, requires that integrated teams of health care providers to treat the entire person, address poly-pharmacy and actively involve patients in care decisions. HIV care delivery has usually focused on only HIV management. This simplistic approach is now morphing into a complex clinical management challenge, where economic factors are magnified significantly, posing questions on cost-effective choices. Health-related disparities within the Hispanic community are observed in their disproportionately lower access to healthcare. Data shows that AIDS-related mortality among US Hispanics has remained constant since 2000, while there has been a concurrent overall decline in AIDS-related deaths (Avert, 2013). Social-structural factors have been implicated in such HIV disparities facing Hispanics, including English-language skills, cultural values, immigration, income inequality, and substance use. These factors fuel stigma/discrimination that constitute barriers to HIV treatment and prevention within the Hispanic community.

Aging minority adults with HIV will require an extensive combination of private and public resources as well as other supports to achieve optimal health outcomes. Because health disparities persist across individuals, policy-makers have devoted greater attention to the HIV care continuum since choices to maximize societal resources must be made given mutually exclusive alternatives related to scarcity in health care supply.

Rigorous HIV treatment adherence is unique among medication regimens for chronic diseases. As the burden of disease (multi-morbidity) increases for the older HIV-positive population, the need to achieve optimal medication adherence is an utmost priority. The most consistent predictor of ART non-adherence and medication non-adherence is depression. Unmanaged depressive disorders occur in a majority of HIV infected patients. In HIV patients, a consistent link is observed between depression and low ART adherence (p<0.0001) with even mild depressive symptoms correlated with low ART adherence (Gonzalez, Batchelder, Psaros, & Safren, 2011). Multiple studies show that people with HIV do respond to typical anti-depression treatments, yet high levels of depression persist in all HIV populations, including older adults.

## 2. Literature Summary on Aging and HIV Among Older Hispanics

Latinos have a 44% increased risk for major depression relative to Whites, and older Latinos are more likely to present with clinically significant depressive symptoms compared with older Whites (Dunlop et al., 2003; Falcon & Tucker, 2000). Literature and meta-analytic analyses find that Puerto Ricans have consistently higher rates of depression compared to Mexican Americans; with the relative position of other groups (Cubans, Dominicans) regarding depression is equivocal (Alegria et al., 2008; Guarnaccia, 2002). Rates of depression among older Latinos may be higher compared with the general population. For example, among Puerto Rican adults age 50 and older, 34% to 61% screened positive for significant depressive symptomatology while 12% met the DSM-IV criteria for major depression (Robinson, Gruman, Gaztambide, & Blank, 2002). Sociocultural factors that have been linked to depression among older Latinos include levels of Anglo acculturation, social isolation and support, family issues, and caregiver burden (Aranda, 2001; Guarnacia, 2002; Robison et al., 2003). When assessing gender differences, Latinas with HIV are often isolated and report depression and suicidal ideation (Enriquez et al., 2010). In studies of sadness and perceived treatment helpfulness, Latinas had significantly higher depressive rates among when compared to males (Lorenzo-Blanco & Delva, 2012).

Substance use, histories of childhood and/or adult violence, and familism (i.e., the primacy of the family over the individual) have been associated with depression among Latinos with HIV (De Santis, 2012). Depression becomes a more significant variable for health care delivery due to the pervasive stigma around mental health issues in Latino communities, that are more likely to perceive persons with mental illness to be dangerous compared to other ethnic groups (Whaley, 1997). This reflects findings that Latinos have significantly higher levels of shame and embarrassment surrounding mental health compared with non-Hispanic Whites (Jimenez et al., 2013). Other barriers for Latinos to behavioral health care include transportation difficulties, employment issues, patient-provider issues, and immigrant documentation (Jimenez et al., 2013). Latino Medicare beneficiaries are half as likely as others to receive treatment for depression and are the least likely to be treated using verbal therapy (Crystal, Sambamoorthi, Walkup, & Akıncıgil, 2003). Latinos with HIV are less likely to follow-up on referrals to mental health services compared to Whites (Seller et al., 2011) and, among Latino emergency department patients who have a high prevalence of depression, many do not remain in behavioral care due to a lack of readiness to seek care. These negative perceptions of mental illness reflect the impact of HIV/AIDS based stigma (Well et al., 2013).

A better understanding of how HIV disease affects older adults, particularly minority groups across age, sexual orientation and race/ethnicity is needed. That data can be used to manage existing or develop new systems for the delivery of health care and supportive services for older adults with HIV. These delivery systems will need to address inherent barriers to achieving optimal heath care outcomes. Such findings/data will be the basis for assessing marginal units of health outcome. To measure disparities across individuals, we used the Oaxaca Decomposition approach to assess physical health (CD4 counts) and mental well-being (depression) disparities experienced by Hispanics in a cross-section of HIV-positive adults age 50 and older.

## 3. Methodology

The theoretical model used in this paper is an adaptation of the Oaxaca-Blinder Decomposition, whose analysis focuses on the domain of health inequalities (Jann, 2008). These methods developed in the 1970’s were based on Oaxaca’s (1973) seminal paper analyzing differences in male-female wage differentials in urban labor markets. The methodology is frequently applied to study differences that occur due to inequalities arising from gender, race or ethnicity. The literature on health disparities has been more often used for assessment of the explained and unexplained factors of income inequality between groups. Decomposition methods evolved beyond the scope of economics and have been applied in other disciplines to analyze health disparities. This study examines two groups, G1= {Whites, Blacks and Others, i.e., non-Hispanics} and G2= {Hispanics}. The outcome variables in two different equations are CD4 counts and depression as measured with the Center for Epidemiological Studies Depression Scale (CES-D). The predictors are social, structural and economic determinants of HIV such as age, education, employment status, HIV and AIDS diagnoses as well as comorbidities.

The goal of using this approach is to account for changes in health status, measured by CD4 cell counts and levels of depressive symptoms, between G1 and G2 as a function of a set of factors that vary systematically with socioeconomic status (Equations 1 to 3). Our methodology helps identify gaps in the mean outcomes between Hispanics and Non-Hispanics. The basis for our method is summarized as follows:


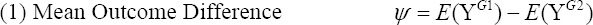



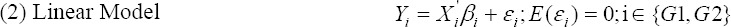



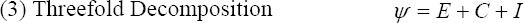


Thus, X is a vector containing a set of explanatory variables and a constant term, the β coefficients include the slope parameters and intercept and ε is the stochastic error term. Our analysis shows the presence of HIV-related disparities between Older Hispanics and Older Non-Hispanics through the relationship in the threefold decomposition (Equation 3). In the explained factor or endowments effect (Equation 4), we account for the differentials due to group inequalities in the predictors (age, education, HIV/AIDS diagnoses and income status), evaluated by the coefficients (β’s) of Hispanics. For the unexplained factor or coefficients effect (Equation 5), we measured the contribution of differences in the estimates or coefficient, which are weighted by the expected value of the explanatory variables (age, education, HIV/AIDS diagnoses and income status) for Hispanics. Finally, the interaction effect (Equation 6) expounds that differences in the coefficients and predictors are present among Hispanics and Non-Hispanics at the same time. Our empirical model encompasses these three components:


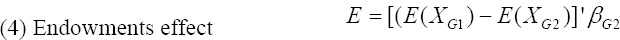



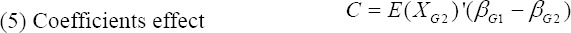



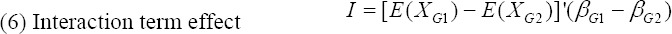


In our econometric model, the predictors assist not only in identifying the overall discrimination effect, but also allows for the quantification of the individual impact of socioeconomic and epidemiologic factor affecting HIV rates among older adult. In addition to the analysis of the total decomposition of the outcome differentials (yNH - yH), the contribution of individual elements in the right-hand side of Equation 2 is worth studying (Jann, 2008).

## 4. Data and Stylized Facts

We used data from the Research on Older Adults with HIV (ROAH) study, a comprehensive research database of 1,000 older adults with HIV living in New York City, NYC (Karpiak, 2006; Brennan, 2009). This survey used instruments grounded on standardized tests to account for demographics, health status, sexual behaviors, social networks, stigma and psychological resources. Based on self-reported health, the ROAH study addresses the burden of the NYC healthcare system and its ability to meet the increased demands of older adults with HIV. It provides information on how limited resources are allocated to determine the needs of older adults with HIV. ROAH is helpful in identifying differences in the needs of groups across gender and race/ethnicity, as well as defining those psychosocial factors such as stigma that contribute to differences in health outcomes (Karpiak, 2006; Brennan, 2009).

The target population for our study are individuals with HIV, who are 50 years and older defined by two racial/ethnic groups: Hispanics (case) and Whites/Blacks/Others (control). The composition of Hispanics varies across cities and the extant 2005–2006 data shows that in NYC the three primary Hispanic origin groups are Puerto Ricans, Dominicans and Mexicans. NYC represents a prime laboratory to assess the burden of disease in specific subgroups, particularly with regard to HIV, due to its social stratification and differentials in exposure to diseases (DelaCruz, 2013). In 2010, 77% of people living with HIV in NYC were older than age 40 and 42% were over age 50. To date, AIDS-related mortality remains high.

ROAH explores the nature and adequacy of health support linkages available to older HIV-positive individuals and is the largest comprehensive study of the emerging aging HIV population. Its variables included basic demographics, health parameters, social networks, access to care, depression, stigma, loneliness, substance use, sexual behavior and spiritually. In ROAH, 70% were men, 29% women and 1% transgender with 50% Black, 33% Hispanic, 13% White and 4% other races. The sample characteristics reflect closely with New York City HIV/AIDS epidemiology data (NYCDOH Surveillance Data 2005). One fifth of the participants were foreign born and our sample shows that 40% of all households are comprised of Latinos born outside the US. In ROAH, Hispanics were more likely to be foreign-born compared with Whites or Blacks.

In ROAH, half of the participants reported a concurrent diagnosis of HIV and AIDS. An AIDS diagnosis indicates that HIV disease has progressed to a stage where immune system function is at or near collapse. Although ART drugs can reverse this status, having an AIDS diagnosis is associated with increased risk of morbidity and mortality as the person ages. CD4 cell counts are a bio-measure of immune system function. People who are not HIV-infected typically evidence CD4 counts in the 1000-1200 range. The higher the CD4 cell count, the higher the functionality of the immune system. Below 200 CD4 T-cell counts the person is at high risk for AIDS. An individual must adhere to ART 90% of the time to sustain immune function and relatively good health; otherwise, the immune system builds resistance to medication.

## 5. Results

Our findings show the Oaxaca decomposition examining health disparities associated with two different health outcomes, immune-compromised individuals and depressive symptoms (y’s), between older Non-Hispanics (G1) and older Hispanics (G2), while controlling for specific environmental factors that influence the path of the HIV epidemic in NYC (x’s). All the calculations were performed in STATA 13 (see Appendix for a description of the variables in the empirical equations). Tables 1(a) and 1(b) establish the threefold breakdown of the Oaxaca Decomposition, which explains how much of the overall gap is attributable to the explained factor (effect of the independent variables) rather than differences in the unexplained component (effect of the estimators or β’s). The decomposition analysis also includes an assessment of the interaction term.

**Table 1:** The Oaxaca Decomposition Regression: Non-Hispanics (G1) and Hispanics (G2)

***Table 1a**. Output from CD4 Counts*	***Table 1b**. Output from Depression*
Mean prediction high (H): 485.85	Mean prediction high (H): 21.9
Mean prediction low (L): 423.91	Mean prediction low (L): 20.1
Raw differential (R) {H-L}: 61.94***	Raw differential (R) {H-L}: 1.80 *
• Due to endowments (E): -434.37	• Due to endowments (E): -0.40
• Due to coefficients (C): 68.13***	• Due to coefficients (C): -0.96
• Due to interaction (CE): 428.18	• Due to interaction (CE): -0.41

*** p < 0.01

p < 0.05**

* p < 0.10.

The focus of this analysis is to explain the gap in the mean y’s, for which the raw differential of 61.94 in CD4 counts and a raw differential of 1.80 in depression symptoms occurs among the two groups, Non-Hispanics and Hispanics. At the standard position, the two groups have different levels of depression and CD4 counts. Having a stronger immune system and better mental health are preferred and, as expected, Non-Hispanics have an advantageous position at the baseline and increases at every possible x’s value. For CD4 counts (Table 1a), the high mark belongs to Non-Hispanics and the low mark corresponds to Hispanics. For depression measurements (Table 1b), the higher value resembles CES-D scores for Hispanics and the low for Non-Hispanics.

In Table 1a, the group differences in the predictors weighted by the coefficients for G2 (endowment effect of 434.37 in absolute numbers of CD4 levels) show the expected change in Hispanics’ health status, as if they had non-Hispanics’ predictor levels. The increase of 434.37 indicates that divergences in characteristics between the two groups accounts for a large quantity in the immune-suppression gap. Our model also captured the differences in the estimates weighted by the predictors for G2 (coefficient effect of 68.13 in CD4 counts), which quantifies changes in CD4 cells if Hispanics had the coefficients matching the control group. If Hispanics experienced the same coefficients (β’s) as Non-Hispanics, their T-cell levels would increase by 68.13 points, almost approximating the levels for the control group. Finally, we were able to observe the interaction effect (values of 428.18 in immune-suppression) describing concurrent variances in endowments and coefficients between G1 and G2, expressed by a steeper and diverging health-response curve for Hispanics compared to other ethnicities. A high score of the interaction term in the CD4 cells coefficient suggests that a large amount of the decomposition is the result of the interaction effect.

Accordingly, Table 1b) explains an endowment effect of -0.40 in the CES-D value and shows the expected change in Hispanics’ depression metric, if they had non-Hispanics’ predictor levels. Although the difference in depression levels between Hispanics and Non-Hispanics is sizable, the absolute value of 0.40 suggests that dissimilarities in endowments account for a relatively small amount of difference in the mental health gap. Our regression also described the disparities in the estimates weighted by the predictor for G2 (coefficient effect of -0.96 in CES-D markers), quantifying changes in depression if Hispanics had the coefficients matching the control group. If G2 had the same estimators (β’s), depression would decrease by almost 1 point in the CES-D scale, thereby reducing the gap between the case and the control groups. Lastly, the interaction effect is negligible with a value of -0.41 for depression, which indicates that the slopes of the curves for both groups’ equations are almost parallel to each other.

**Figure 1:**
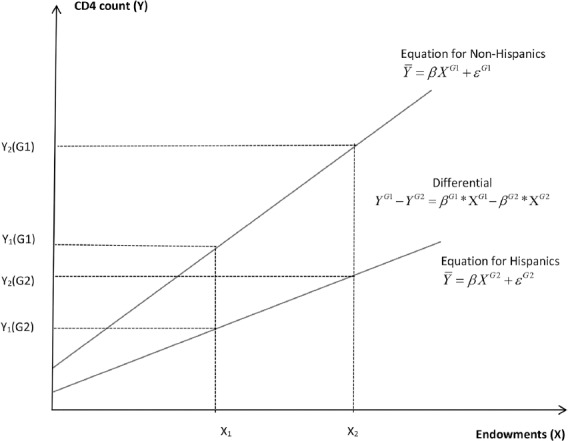
Oaxaca Decomposition: CD4 count levels

At each value of the predictors (x’s), the outcome variable (T-Cells or depression) is always better for the more advantageous group, which in our study is represented by Non-Hispanics. A graphic depiction of the estimates for CD4 count levels between our two different groups is shown in Figure 1 above, illustrating the two group’s predicted T-Cell count means at the baseline and showing a steep difference in CD4 counts between the case and control groups. This suggests that Hispanics’ immune response is 13% lower relative to non-Hispanics, which is sizable and significantly different from zero at the 1% level. This difference may be due to the endowments and factors determined outside the healthcare system. As anticipated, G1 has a more beneficial fitted line than G2 and the expected value of health status is better for non-Hispanics. The slope coefficient measures the level of differentials in health outcomes between G1 and G2 and is explained by the disparity in the predicted means estimated by the regression lines.

**Figure 2:**
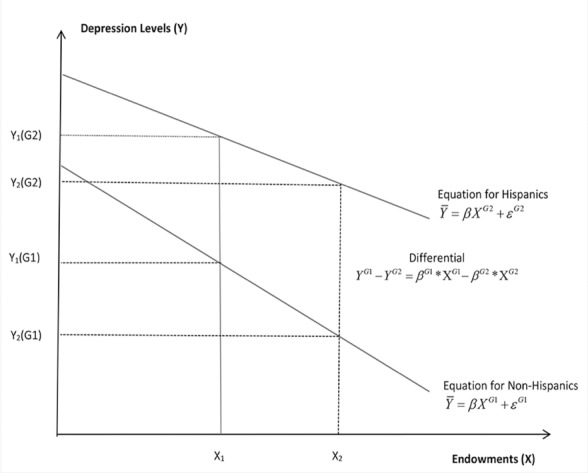
Oaxaca Decomposition: Depressions levels

Figure 2 below portrays the additive effects of estimates for depressive symptoms between our two groups with a value for health disparities of 1.80 at the 10% significance level, indicating that depression is more severe for Hispanics (21.9) than for Non-Hispanics (20.1). The expected mean value of depression is 8% higher for Hispanics, which may explain lower adherence and higher risk of developing full blown AIDS. The regression line shows an inverse association between depressive symptoms and endowments, suggesting a greater chance of ART non-adherence among Hispanics, leading to higher depression levels compared to non-Hispanics.

Tables 3 and 4 (see appendix) determine to what extent the gaps of individual independent variables contribute to the overall explained disparity. Differences between Hispanics and Non-Hispanics are augmented by socioeconomic factors such as education and income status as well as epidemiological determinants that include likely contributing the length of time since the HIV and/or AIDS diagnoses. In both expected outcomes (CD4 and depression), age seems to contribute to greater differences in their means, disfavoring disproportionately Hispanics.

## 6. Discussion: Policy Implications and Final Comments

To analyze HIV disparities between Hispanics and Non-Hispanics, we used assessments of declining health and depression as health outcome indicators. Older Hispanics are disadvantaged due to factors associated with social and structural determinants of health. Our findings identify a significant factor that contributes to health disparities in older Hispanics with HIV in NYC. That factor was quantified and classified as health disparities by the Oaxaca Decomposition model. These findings identify additional health disparities for Hispanics who are growing older with HIV. Given the well-documented links between CD4 levels and multi-morbidity, as well as ART non-adherence and depression in older HIV populations (Havlik, 2011), special efforts must address these disparities among older HIV-positive Hispanics. In fact, the provision of ART has not been uniform among different racial/ethnic groups.

There is an urgent demand for more analysis to study the structural and social determinants of the HIV disease among older Hispanics and other vulnerable groups to optimize societal resources and thereby better resolve health-related gaps. Assessing disparities and the cost per unit of health outcome at the margin will provide a guide for measuring the quality and quantity of life gained due to appropriate interventions. Since Hispanics in the ROAH study were all English-speaking, (the sample neither includes those who speak Spanish only nor captures those assimilated into the mainstream culture), the disparities found in this paper likely represent a conservative estimate of the discrimination barriers faced by the broader HIV-positive older Hispanic community.

The majority of health and civil society organizations cite a lack of bilingual and culturally sensitive HIV and AIDS services as a key obstacle for the city’s Hispanic HIV epidemic. Culturally appropriate policy and program initiatives that increase HIV testing rates, encourage routine/frequent sero-status assessment, and improve sustained connection to care and treatment adherence, will result in higher CD4 counts and lower risk for multi-morbidity.

## Appendix

Description of the Variables:

Left-hand-side (dependent) variable:

- CD4 = recent CD4 cell count: (range, (5-1200), lower CD4 represents lower immune function to 2000)
- CES-D = depression scale: (range, (0 to 52), higher numbers indicate higher levels of depression)

Right-hand-side (independent) variables:

- AGE = years of age.
- HIVDIAG = the number of months since diagnosis.
- AIDSDIAG = diagnosed with aids (yes or no).
- LHS = less than high school education (level).
- HS = high school education (level).
- SC = some college education (level).
- C = college (level, base or reference variable).
- WORKING = are you presently working (yes or no).
- UNEMPLOYED = are you presently unemployed (yes or no).
- DISABILITY = are you presently on disability (yes or no, base or reference variable).
- ILLTOT = Number of Comorbid Health Conditions.

**Complementary Tables**

**Table 2:** Oaxaca Decomposition Results CD4 White Cell Count Levels

Groups	Mean Difference	Endowment (E)	Coefficient (C)	Interaction (C*E)
All Others – Hispanics	61.94***	-434.37	68.13***	428.18
Whites – Hispanics	10.1	-24.9	-7.32	42.32
African Americans – Hispanics	68.26***	-573.86	60.69**	581.16

*** p < 0.01

** p < 0.05

* p < 0.10.

**Depression Levels**

**Table:**

Groups	Mean Difference		Endowment (E)	Coefficient (C)	Interaction (C*E)
All Others – Hispanics		-1.79*	-0.4	-0.96	-0.41
Whites – Hispanics		1.08	-1.3	-59.38	-57
African Americans – Hispanics		-2.27**	-0.26	-1.70*	-0.31

p < 0.01***

** p < 0.05

* p < 0.10.

**Table 3:** Oaxaca Decomposition Cd4 White Cell Count Levels a) Hispanics and Non-Hispanics

	Hispanics			Non-Hispanics		
	
CD4 Levels	Coef.	SE	P>|t|	Coef.	SE	P>|t|
**Mean Prediction**	423.91			485.85		
Age	-1.09	4.3	0.799	1.7	3.44	0.62
Working	115.39	55.13	0.038**	30.65	45.89	0.505
Unemployed	58.45	41.08	0.156	89.28	34.82	0.011**
Less High School	5.05	47.27	0.915	65.69	45.79	0.152
High School	-16.6	45.69	0.717	42.46	40.49	0.295
Some College	23.99	51.6	0.642	-6.2	39.02	0.874
AIDS Diagnosis	-196.71	32.44	0.000***	-0.1	0.29	0.731
HIV Diagnosis	-0.52	0.26	0.051*	-0.39	0.13	0.003***
Illot	4.74	7.18	0.51	-1.36	0.82	0.817
_Cons	639.27	248.98	0.011**	413.92	201.39	0.040**
						

b) Whites and Blacks Only						

	Whites			Blacks		
	
**CD4 Levels**	**Coef.**	**SE**	**P>|t|**	**Coef.**	**SE**	P>|t|

**Mean Prediction**	434.01			492.17		
Age	4.42	6.58	0.504	-0.29	4.18	0.944
Working	11.07	65.51	0.866	28.21	61.24	0.645
Unemployed	32.8	91.76	0.722	106.1	40.46	0.009***
Less High School	-138.52	147.84	0.352	83.16	60.27	0.169
High School	-33.82	72.48	0.642	54.63	56.61	0.335
Some College	-91.99	64.46	0.158	18.21	55.11	0.741
AIDS Diagnosis	-80.62	59.44	0.179	-0.07	0.31	0.814
HIV Diagnosis	-0.24	0.25	0.339	-0.37	0.17	0.030**
Illot	-1.89	11.05	0.865	-3.07	7.27	0.673
_Cons	323.02	389.49	0.41	502.9	244.73	0.041**

*** p < 0.01

** p < 0.05

* p < 0.10.
